# First Clinical Experience with a New Device for the Removal of Cochlear Schwannomas

**DOI:** 10.3390/jcm13113300

**Published:** 2024-06-03

**Authors:** Christoph J. Pfeiffer, Conrad Riemann, Rayoung Kim, Lars-Uwe Scholtz, Matthias Schürmann, Ingo Todt

**Affiliations:** Department of Otolaryngology, Head and Neck Surgery, Medical School OWL, Bielefeld University, Campus Mitte, Klinikum Bielefeld, 33604 Bielefeld, Germany; christoph.pfeiffer@klinikumbielefeld.de (C.J.P.); conrad.riemann@klinikumbielefeld.de (C.R.); rayoung.kim@klinikumbielefeld.de (R.K.); lars-uwe.scholtz@klinikumbielefeld.de (L.-U.S.); mathias.schuermann@klinikumbielefeld.de (M.S.)

**Keywords:** cochlear implant, intra-cochlear schwannoma, intralabyrinthine schwannoma, tissue removal device

## Abstract

**Background**: In most cases, intralabyrinthine schwannoma (ILS) occurs in patients with unilateral hearing deterioration or neurofibromatosis type II (NF II). The pattern of localization of these tumors varies but mostly affects the cochlea. Extirpation of the cochlear schwannoma, if hidden by the cochlea modiolus, is difficult under the aspect of complete removal. Therefore, a tissue removal device (TRD) was designed and tested in temporal bones. The principle of handling the new device is a pushing and pipe cleaner handling inside the cochlea. This present study aimed to describe the first in vivo experience with the newly developed TRD for removing cochlear intralabyrinthine schwannomas. **Methods**: In three patients, the TRD was used for the tumor removal of cochlear schwannomas. In two patients with a cochlear schwannoma in combination with a cochlea implantation and one patient suffering from NF II, a cochlear schwannoma was removed with the TRD. The access was performed with a posterior tympanotomy, an enlarged round window approach and an additional second turn access. The device was inserted and extracted gradually from the second turn access until the rings were visible in the second turn access. By pushing and pipe cleaner handling, the tumors were removed. An MRI control was performed on the day postoperatively with a T1 GAD sequence. **Results**: Tumor removal with the TRD was performed in a 15-min procedure without any complications. An MRI control confirmed complete removal on the postoperative day in all cases. **Conclusions**: In vivo handling of the device confirmed straightforward handling for the tumor removal. MRI scanning showed complete removal of the tumor by the TRD.

## 1. Introduction

The intralabyrinthine schwannoma (ILS) is a well-known tumor of the lateral skull base occurring in various locations. They are most frequently found in the cochlea with a rate of about 60% [1,2]. Cochlear implantation is the well-accepted form of rehabilitation for hearing loss in these cases and was first described by Kronenberg [3].

Related to the broadening of indication for cochlear implantation to the group of patients with single-sided deafness, the number of found ILS is increasing, which is underlined by a growing number of international publications [4].

The handling of the tumor itself is controversial in its discussions. The mean growth rate of the tumor is calculated as 0.25 mm per year [5] and hence is considered as low. However, the fact that the tumor does enlarge and that single faster-growing cases are known contributes to the relevance of the debate revolving around the management of this tumor. The approaches are quite diverging. Some describe a cochlear implant insertion laterally, through the tumor without its removal [6,7,8] or after radiation [6], but most groups extirpate the tumor before array placement. The surgical handling applied depends on the localization of the tumor. Should the tumor be covered by the modiolus, different techniques are known, like a cochlectomy [9,10], which includes revealing the top of the promontory, removal of the tumor, and covering the situs with cartilage after electrode placement. Another technique is the application of a Cochlear Advance Dummy electrode for an extended round window access and pushing the tumor through a second turn access [11]. A third technique consists of pushing dry gel-foam into the extended round window access and by this pushing the tumor out of the second turn access. Floating, upswelling of the gel-foam enables removal by sucking the material out of the cochlea [12]. A fourth technique uses a dummy electrode by cutting the bulky basal part and pushing it in a turned manner to push the tumor out of the second turn access. A fifth technique is close to the second technique but uses a 28 mm stiffened lateral wall device (TRD), but until now, it has just been demonstrated in a human temporal bone model [13]. This technique showed that it is easy to grab and remove the tumor by a by pipe-cleaner-like technique.

Besides these surgical techniques, watch-and-wait or radio-surgical treatment approaches have been discussed and performed, especially in cases of NF II [7].

As shown for central tumors, the MRI-based follow-up is highly important and well executable, even in cases with a cochlear implant, since the cochlear implant magnet artifacts are no longer an obstacle [12]. More precisely, implant and head positioning in the scanner, choice of MRI sequence, and implant magnet have been shown to allow a follow-up of VS [12], ILS [14], and glioma [15] by MRI.

The present study aimed to describe the first set of experiences of a newly developed device for removing cochlear intralabyrinthine schwannomas in vivo.

## 2. Material and Methods

Three patients (3 female, age: 45; 55; 63) who underwent cochlear schwannoma removal with a new TRD were included in this retrospective study. One patient is suffering from an NFII (a cochlear schwannoma with combined persisting VS), and two are with a pure cochlear schwannoma and an additional single-staged cochlear implantation. Initial symptoms for all patients were progressive hearing loss followed by an MRI which confirmed the cochlear tumor. All patients suffered from unilateral deafness at the time of surgery. All patients showed unilateral vestibular receptor dysfunction (cVEMP, caloric testing) without apparent clinical symptoms.

**Figure 1:**
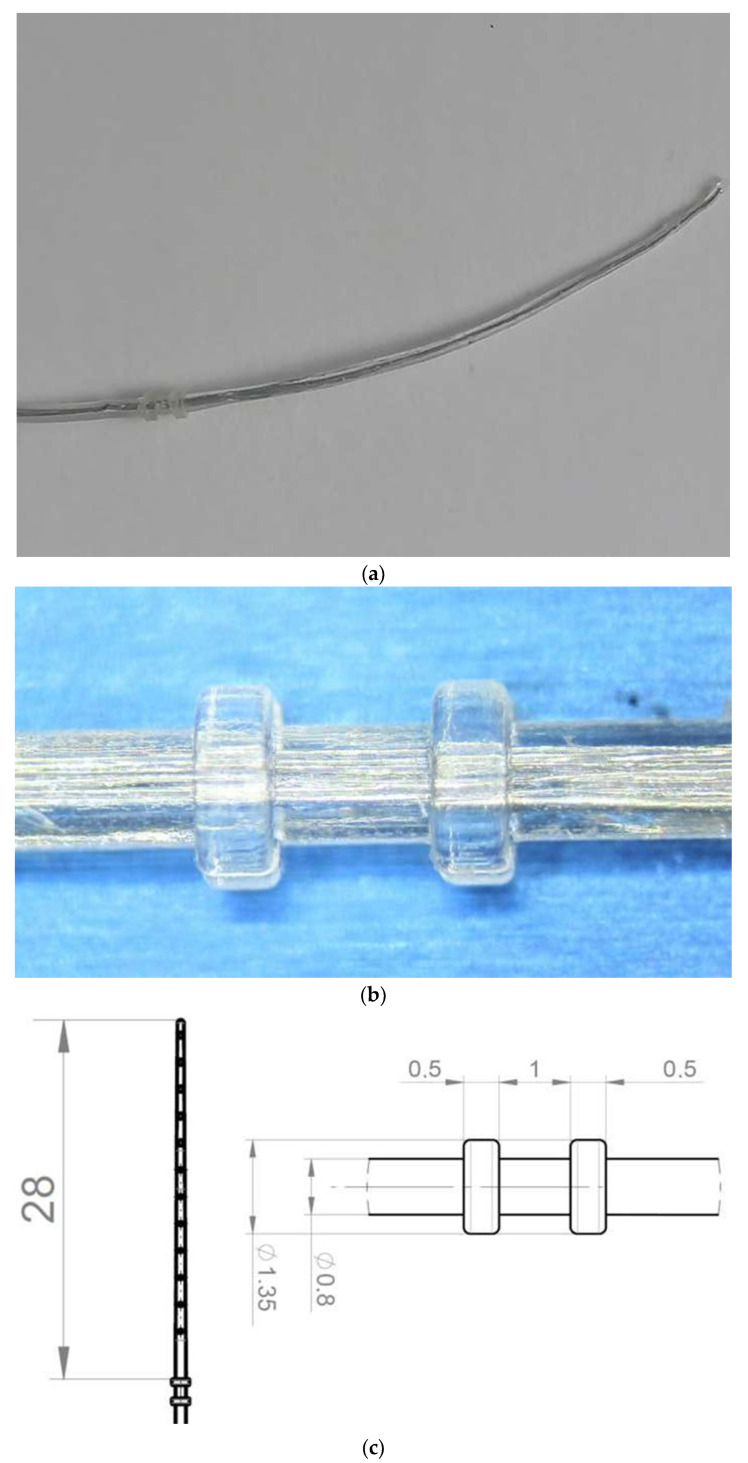
(**a**–**c**): Electrode design.

The used tissue removal device (TRD) had the outer shape of a CI electrode array. It is a custom-made device by MEDEL Company (Innsbruck, Austria). It is mechanically stabilized, and two silicone rings are basally added with a diameter of 1.35 mm. These silicone rings are 1 mm apart and 0.5 mm in width (Figure 1a–c) [13].

On the day after surgery, an MRI scan was performed to evaluate residual tumor persistence with a 3T MRI scanner (Philips, Achieva, Amsterdam, The Netherlands) by applying the T1 KM sequence.

This study was approved by the Ethical Committee of the University of Münster/Bielefeld (2023 689 f-S; 11.04.2024).

## 3. Results

Intraoperative findings confirmed the MRI scan of a schwannoma in the first and second turn for all the cases.

In all the cases, a regular surgical procedure to remove cochlear ILS through a posterior tympanotomy after removal of the incus was performed. An enlarged cochleostomy was burred, and additional access through the posterior tympanotomy to the second turn of the cochlea was introduced about 1.5 mm caudally from the facial nerve and 1 mm above the oval window, as described by others.

**Figure 2:**
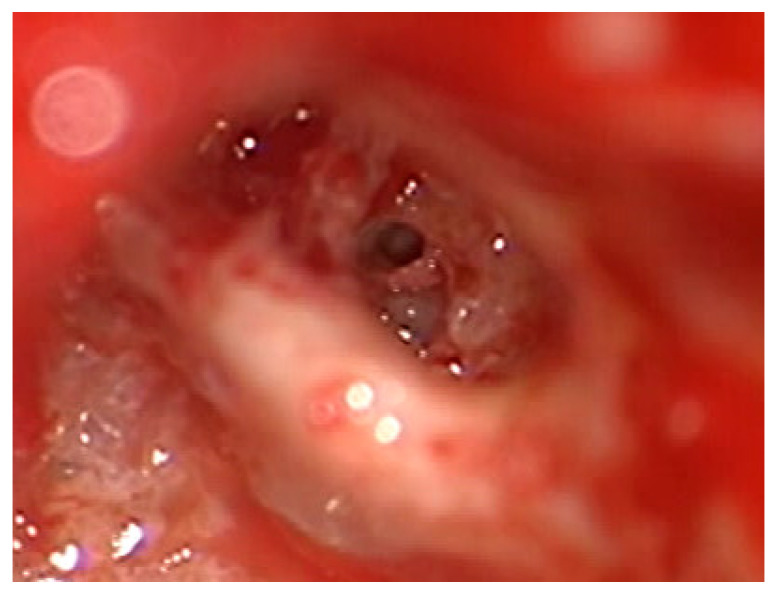
Tumor in the scala tympani with a basilar membrane and scala vestibuli.

**Figure 3:**
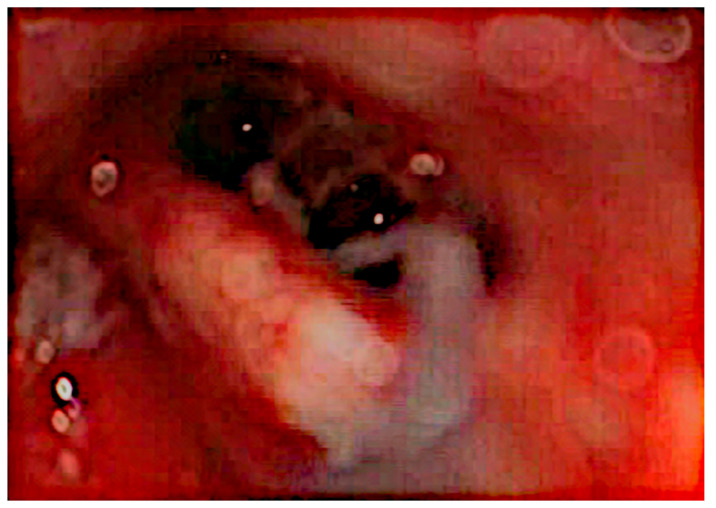
Introduction of the TRD.

**Figure 4:**
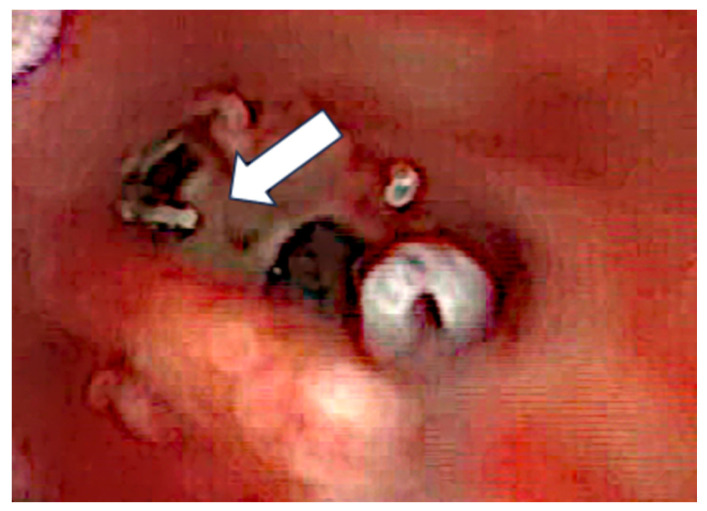
Tip visualized in the second turn (arrow).

**Figure 5:**
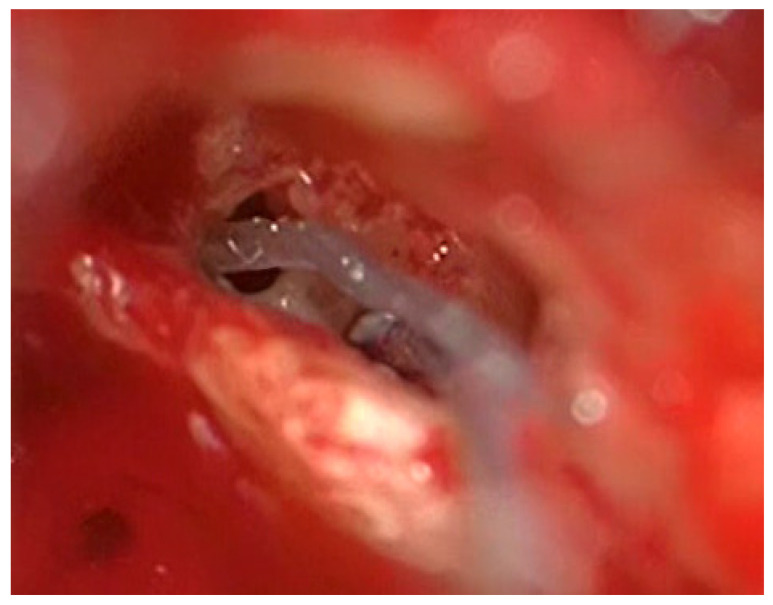
Pipe cleaner handling of the TRD.

**Figure 6:**
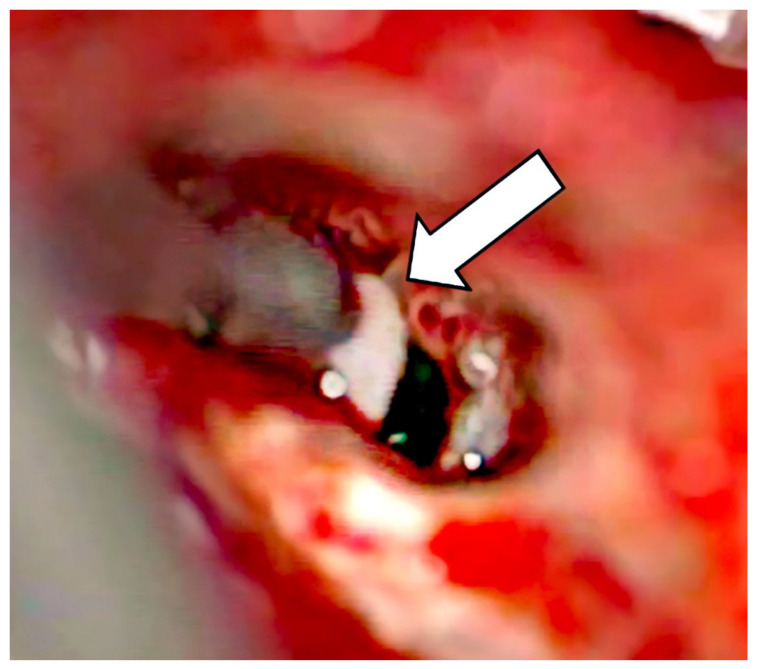
Removal of tumor parts (arrow).

After the removal of parts of the tumor through the enlarged cochleostomy (Figure 2), the TRD was introduced (Figure 3) until the tip was visualized (Figure 4) and carefully grabbed out of the second turn access. By grabbing the tip, the device slipped into the cochleostomy and pushed parts of the tumor out of the second turn. Residual tumor parts could be detached by pipe cleaner handling (Figure 5) and sucked out (Figure 6).

**Figure 7:**
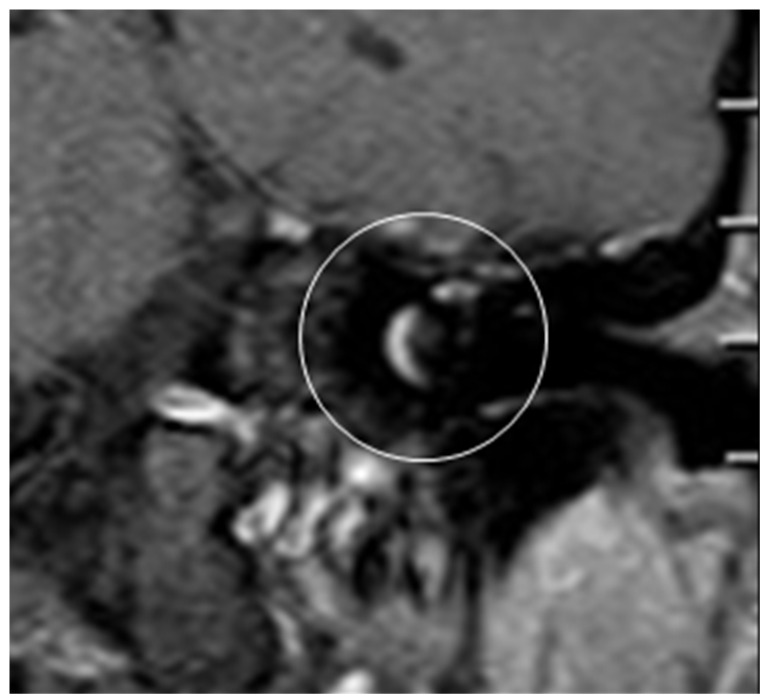
Pre-OP MRI.

**Figure 8:**
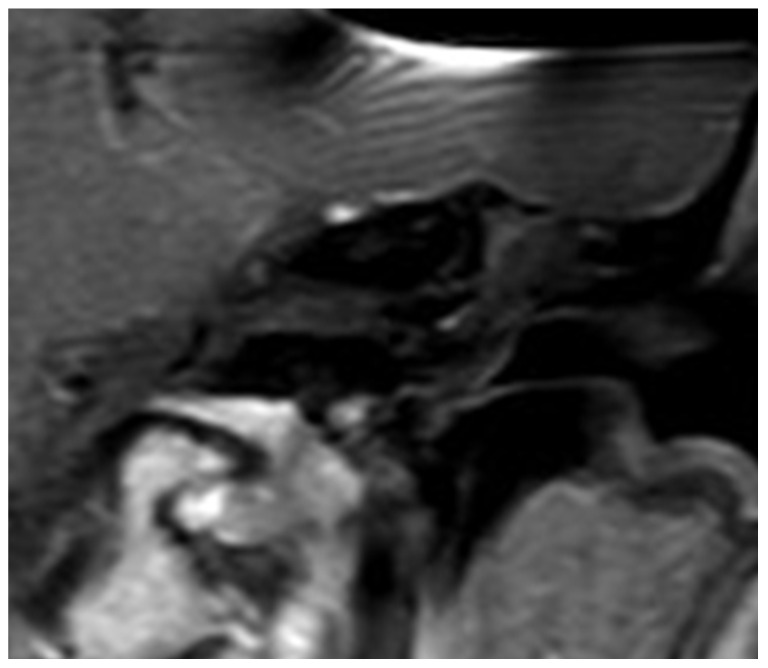
Post-OP MRI.

The whole procedure lasted 15 min in each case and could be performed without any complications. The second turn access was closed with a large piece of fascia. Intraoperative impedance measurements showed regular slightly elevated values for case 1 and 2. ECAP thresholds were slightly elevated, and the responses could be measured for all contacts for case 1 and 2. Histology confirmed the intraoperative assumption of a schwannoma. A genetic testing was not performed. Comparing pre-OP (Figure 7) and post-OP (Figure 8) MRI scanning confirmed a complete removal of the tumor. The individuals’ data confirmed that, if the schwannoma is limited to the cochlea turn, regular speech understanding can be achieved (Table 1).

## 4. Discussion

Intralabyrinthine schwannomas are tumors of the lateral skull base that can occur as part of an NFII or spontaneously.

In many cases, the accompanying hearing loss is restored by cochlear implantation. Apart from the concept of non-removal of the tumor applied in some centers by pushing the cochlear implant electrode through the tumor [6,7,8], the ILS is mostly removed to prevent the risk of tumor growth. Recently, we described a new device for the removal of cochlear schwannomas [13] in human temporal bone models.

The proposed handling of the device is now verified in the first three in vivo observations. Similar to the observations made in the temporal bone model, the tumor could be removed by a combination of an enlarged cochleostomy and a second turn access. The proposed pushing and pipe cleaner handling by grabbing the tip allows a straightforward performance of the removal. In both cases, the whole procedure of tumor removal lasted 15 min, and the following MRI scan confirmed a complete removal of the tumor without any complications.

Comparing this technique with others, it is important to mention that the audiological outcome of cochlear implantation in ILS cases allows a regular speech audiometric outcome in most cases [16]. Therefore, the handling, time, ease of procedure, and completeness of removal are in focus. Watchful waiting might be an option in cases of functional hearing. But in cases of functional deafness, hearing rehabilitation by a cochlear implant is the treatment of choice. Radiosurgery can be assumed to cause granulation or scar formation, which might complicate a cochlea implantation.

While a cochlectomy is a time-consuming procedure [9,10], it is indicated in cases which need broader visual access to the situs than a pipe cleaner procedure. Comparing the proposed TRD handling with the usage of a Cochlear Advance dummy electrode [11], which is quite close to our handling, the latter electrode is in our experience bulkier and softer, and the tip is more difficult to grip. The smaller size of the TRD positioned laterally in the cochlear scala enabled it to slip out of the second turn access; we assume this procedure to be less traumatic.

The described use of dry gel-foam to push the tumor out of the second turn of the cochlea is, compared to the proposed technique, time-consuming and therefore less favorable [12].

The general question of removing or not removing these tumors can easily be answered by the following question: why not remove it, if one can minimize the risk of a growing tumor by a swift procedure? Future experiences will show if this array can be used even in cases of cochlea granulations apart from schwannomas. Here, cases of intracochlear granulation after the removal of electrodes can be assumed.

The limitations of this study are the small number of patients, which is related to the low occurrence rate of these tumors, and the short follow-up period with the MRI scan. Since these tumors are regularly monitored, we will be able to compare different techniques in terms of their tumor control in the future.

## 5. Conclusions

In vivo, the handling of the TRD confirmed a straightforward handling for cochlear schwannoma removal. Early MRT scanning showed in all cases a complete removal of the tumor by the TRD.

## Figures and Tables

**Table 1:** Individuals’ data.

No.	Age/Gender	Symptom	Location of Schwannoma	Monosylab. One Year with CI
1	45, female	deafness	cochlea	70%
2	55, female	deafness	cochlea	60%
3	63, female	deafness	cochlea and IAC	0%, no CI

## Data Availability

The data used to support this study’s findings are available from the corresponding author upon request.
